# Tissue‐Resident Myeloid and Histiocytic Cells in Health and Disease: Novel Emerging Concepts

**DOI:** 10.1002/ajh.70062

**Published:** 2025-09-12

**Authors:** Peter Valent, Johann Wojta, Petri T. Kovanen, Olivier Hermine, Falko Fend, Karl Sotlar, Hildegard Greinix, Klaus Geissler, Karin Hartmann, Juliana Schwaab, Marco Herling, Laura Boccuni, Lukas Kazianka, Max Vincent John, Wolfgang R. Sperr, Carina Zierfuss, Alexandar Tzankov, Christian Sillaber, Milen Minkov, Gregor Hoermann, Matthew Collin, Hans‐Peter Horny, Torsten Haferlach, Maria Sibilia, Julien Haroche, Paul La Rosée, Alberto Orfao, Michel Arock

**Affiliations:** ^1^ Department of Internal Medicine I, Division of Hematology and Hemostaseology Medical University of Vienna Vienna Austria; ^2^ Ludwig Boltzmann Institute for Hematology and Oncology Medical University of Vienna Vienna Austria; ^3^ Department of Internal Medicine II, Division of Cardiology Medical University of Vienna Vienna Austria; ^4^ Ludwig Boltzmann Institute for Cardiovascular Research Medical University of Vienna Vienna Austria; ^5^ Wihuri Research Institute Helsinki Finland; ^6^ Service d'hématologie, Imagine Institute Université de Paris, INSERM U1163 Centre national de référence des mastocytoses, Hôpital Necker, Assistance publique hôpitaux de Paris Paris France; ^7^ Institute of Pathology and Neuropathology and Comprehensive Cancer Center University of Tübingen Tübingen Germany; ^8^ Institute of Pathology University Hospital Salzburg, Paracelsus Medical University Salzburg Austria; ^9^ Division of Hematology Medical University of Graz Graz Austria; ^10^ Faculty of Medicine Sigmund Freud University Vienna Austria; ^11^ Division of Allergy, Department of Dermatology University Hospital Basel and University of Basel Basel Switzerland; ^12^ Department of Clinical Research University Hospital Basel and University of Basel Basel Switzerland; ^13^ Department of Biomedicine University Hospital Basel and University of Basel Basel Switzerland; ^14^ Department of Hematology and Oncology University Hospital Mannheim Heidelberg University Mannheim Germany; ^15^ Department of Hematology, Cell Therapy, Hemostaseology, Infectious Diseases and Cancer Center Central Germany (CCCG) University Hospital Leipzig Leipzig‐Jena Germany; ^16^ Department of Internal Medicine I, Division of Clinical Oncology Medical University of Vienna Vienna Austria; ^17^ Institute of Medical Genetics and Pathology University Hospital Basel Basel Switzerland; ^18^ Childrens Cancer Research Institute Medical University of Vienna Vienna Austria; ^19^ MLL Munich Leukemia Laboratory Munich Germany; ^20^ Newcastle University Newcastle Upon Tyne UK; ^21^ Institute of Pathology Ludwig‐Maximilian University Munich Germany; ^22^ Center for Cancer Research, Comprehensive Cancer Center Medical University of Vienna Vienna Austria; ^23^ Pitié‐Salpêtrière Hospital, French National Centre for Histiocytoses Sorbonne University, APHP Paris France; ^24^ Department for Internal Medicine II Schwarzwald‐Baar Klinikum Villingen‐Schwenningen Germany; ^25^ Servicio Central de Citometria, Centro de Investigacion del Cancer (IBMCC; CSIC/USAL) Instituto Biosanitario de Salamanca (IBSAL), CIBERONC and Department of Medicine University of Salamanca Salamanca Spain; ^26^ CEREMAST, Department of Hematological Biology Pitié‐Salpêtrière Hospital, Pierre et Marie Curie University (UPMC) Paris France

**Keywords:** dendritic cell neoplasms, histiocytosis, macrophages, mast cells, mastocytosis, monocytes, myeloid leukemia, stem cell neoplasms, stem cells

## Abstract

Although all myeloid cells are considered to derive from hematopoietic stem cells, the cells in each myeloid lineage are heterogeneous populations, and their distribution and functions vary, depending on underlying physiologic and pathologic processes, age, sex, and genetic and epigenetic signatures. In general, myeloid cells can be separated into circulating and tissue‐resident cells. Tissue‐resident myeloid cells can further be divided into cells derived from circulating monocytes, circulating stem cells, or local tissue‐restricted stem or progenitor cells. Depending on underlying diseases and co‐morbidities, the phenotype, function, and distribution of these cells may change substantially. In this article, we discuss new developments in the field and related emerging concepts around tissue‐resident myeloid cells and their role and function in reactive and clonal disorders. Cell types reviewed in depth in this article include monocytes, macrophages, histiocytes, dendritic cells, and tissue mast cells, with a focus on inflammatory disease processes, vascular pathologies, solid tumors, and hematopoietic malignancies. Moreover, the current article provides an update on patient‐related and disease‐related diagnostic and prognostic variables, multi‐parametric prognostic scoring systems, and therapeutic options and algorithms in these neoplasms. Finally, our article provides an overview on the emerging role and impact of precision medicine approaches, translational research, and artificial intelligence in the diagnosis, prognostication, and management of monocytic, histiocytic, and mast cell disorders.

## 1 Introduction

A number of physiologic and pathologic processes are regulated by tissue‐entering and tissue‐resident leukocytes and their products in various organ systems [1, 2, 3, 4, 5, 6, 7]. These cells are key regulators orchestrating the influx, migration, and distribution of other leukocytes and the generation of various inflammatory and tissue‐remodeling molecules, including vasoactive mediators, and growth‐modulating and cell‐activating cytokines and chemokines (Table S1) [1, 2, 3, 4, 5, 6, 7, 8, 9, 10, 11, 12]. Tissue‐resident leukocytes also regulate tissue homeostasis and the immediate and sustained defense against invading microbes and other exogenous harm in healthy individuals and in various pathologic processes [1, 2, 3, 4, 5, 6, 7, 8, 9, 10, 11, 12]. In addition, these cells are involved in tissue remodeling and repair after tissue damage following severe hyper‐inflammatory, infectious, or vascular reactions [3, 4, 5, 6, 7, 8, 9, 10, 11, 12]. Moreover, tissue‐resident leukocytes are involved in the evolution of clinical symptoms in acute and chronic inflammatory, immunologic, and vascular pathologies [7, 8, 9, 10, 11, 12, 13, 14, 15, 16]. Imbalances or deficiencies in tissue‐resident leukocytes may lead to irreversible pathologies, tissue damage, or even death. Finally, tissue‐resident leukocytes and/or their progenitors may acquire somatic mutations and can thereby transform to an overt malignancy [17, 18, 19, 20, 21, 22].

Two groups of tissue‐resident leukocytes have been described: tissue‐fixed lymphoid cells and tissue‐resident myeloid cells. Whereas the function and role of local lymphoid cells in various physiologic and pathologic processes have been well established, little is known about the origin, function, and role of tissue‐resident myeloid cells in various reactive and neoplastic disorders [1, 2, 3, 4, 5, 6, 7, 8, 9, 10, 11, 12, 13, 14, 15, 16].

**FIGURE 1:**
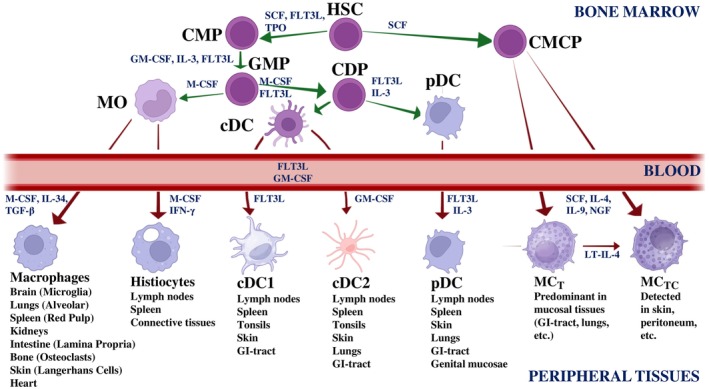
Differentiation pathways and development of tissue‐resident myeloid immune cells from hematopoietic stem cells in healthy tissues. The figure presents a simplified scheme of the differentiation of hematopoietic stem cells (HSC), located mainly in the bone marrow (BM) and blood, into monocytes (MO), macrophages, histiocytes, dendritic cells (DC), plasmacytoid dendritic cells (pDC), and mast cells (MC) in various tissues and organs. Each differentiation step is promoted by specific cytokines. Green arrows represent events taking place in the BM, while red arrows represent the migration of the cells into the peripheral blood (PB), before these cells or their stem or progenitor cells enter various peripheral organ systems. In the BM (or in peripheral organs) the multipotent HSC give rise to common myeloid progenitors (CMP) under the influence of stem cell factor (SCF), thrombopoietin (TPO) and FLT3 ligand (FLT3L). These CMP differentiate into granulocyte‐macrophage progenitors (GMP) under the influence of Interleukin‐3 (IL‐3) and granulocyte‐macrophage colony‐stimulating factor (GM‐CSF). GMP, in turn, give rise to monocytes (MO) and to common dendritic cell progenitors (CDP). Differentiation of GMP into MO is supported by macrophage colony‐stimulating factor (M‐CSF). In several tissues, macrophages arise from MO upon entry, driven by M‐CSF, Interleukin‐34 (IL‐34), and transforming growth factor‐beta (TGF‐β). Specialized macrophages (e.g., Kupffer cells, microglia, and Langerhans cells) are influenced by local factors such as TGF‐β, Interleukin‐10 (IL‐10), or colony‐stimulating factors (CSF). Histiocytes, a type of resident macrophage in tissues, differentiate locally under the influence of M‐CSF and TGF‐β. BM conventional dendritic cells (cDC) arise from CDP, which differentiate from GMP under the influence of GM‐CSF and FLT3L. cDC further mature into cDC1 (dependent on FLT3L) or cDC2 (dependent on GM‐CSF). Plasmacytoid dendritic cells (pDCs) originate also from CDP but require FLT3L and IL‐3 for their development. Finally, committed mast cell progenitors (CMCP) differentiate from BM and circulating HSC in the presence of SCF in various tissues and organs. In these tissues, CMCP differentiate into mature mast cells (MC) in the presence of SCF and other cytokines, such as IL‐4 or IL‐9. In mucosal tissues, MC express preferentially tryptase (T) and are termed MC_T_, whereas in connective tissues, MC express both tryptase and chymase (TC) and are termed MC_TC_ cells. CDC, classical dendritic cells; CDP, common dendritic cell progenitors; CMCP, committed mast cell progenitors; CMP, common myeloid progenitors; FLT3L, FLT3 ligand; GM‐CSF, granulocyte‐macrophage‐colony stimulating factor; GMPs, granulocyte‐macrophage progenitors; HSCs, hematopoietic stem cells; IFN‐γ, interferon‐gamma; IL‐, interleukin; M‐CSF, macrophage‐colony stimulating factor; MC, mast cells; MC_T_, mast cell expressing tryptase; MC_TC_, mast cell expressing both tryptase and chymase; MO, monocytes; pDC, plasmacytoid DC; SCF, stem cell factor; TGF‐ß, transforming growth factor‐beta; TPO, thrombopoietin. The final design of the figure was established by using BioRender. [Color figure can be viewed at wileyonlinelibrary.com]

Tissue‐resident myeloid cells include macrophages, dendritic cells (DC), plasmacytoid DC (pDC) and mast cells (Table 1). Many tissue‐resident myeloid cells are considered to be replenished from CD34+ hematopoietic stem cells in the bone marrow (BM) or peripheral blood (PB), or from circulating monocytes (figure 1, Table 1) [1, 2, 3, 4, 5, 6, 11, 12, 13, 14, 19, 20, 21, 22]. However, some tissue‐resident myeloid cells may originate from yolk sac‐derived progenitors. These cells, such as skin mast cells, Langerhans cells in the skin, or microglia in the brain, have populated relevant tissues before birth and subsequently self‐maintain throughout life under steady state conditions [23, 24, 25, 26].

**TABLE 1:** Tissue‐resident myeloid and histiocytic leukocytes—overview of cells and cell subsets discussed in the conference.

Cell type	Putative origin	Unique subsets^a^
Monocytes	Bone marrow‐derived, circulating, and local hematopoietic stem cells	Classical CD14^++^/CD16^−^ monocyte^b^
		Intermediate CD14^++^/CD16^+^ monocyte^b^
		Non‐classical CD14^++^/CD16^++^ monocyte^b^
Macrophages	Bone marrow‐derived stem cells, circulating stem and progenitor cells; monocytes	M1: classically activated
		M2: alternatively activated (2a–2d)
		Polarized activation stages
		Tumor‐associated macrophages
Histiocytes	Bone marrow‐derived stem cells, circulating stem and progenitor cells; monocytes	Often used as umbrella term for: macrophages and dendritic cells of myeloid origin
Dendritic Cells (DC)^c^	Bone marrow‐derived stem cells, circulating stem and progenitor cells; monocytes	Monocyte‐derived/related DC
		Macrophage‐derived/related DC
		Myeloid Langerhans cell (subsets: DC1, DC2)
		Plasmacytoid DC^d^
Mast cells (MC)	Bone marrow‐derived stem cells and/or local progenitor cells	Tryptase^+^ MC (MC_T_)
		Tryptase^+^/chymase^+^ MC (MC_TC_)

^a^ Major unique subsets are presented; however, even more organ‐specific cell types and subsets have been described.

^b^ All monocyte subsets are defined by expression of CD45 and CD14; but non‐classical monocytes exhibit low amounts of or no CD14.

^c^ Dendritic cells (DC) are defined by their characteristic cytoplasmic extensions.

^d^ Plasmacytoid DC do not express classical myeloid marker antigens.

During the past 3 decades, our knowledge about tissue‐resident myeloid cells in health and disease increased substantially. These cells exhibit unique functional properties, characteristic morphologies, and specific profiles of cell surface and cytoplasmic antigens (Tables 1 and 2) [1, 2, 3, 4, 5, 6, 7, 8, 9, 10, 11, 12, 13, 14, 15, 16]. However, several questions remain about the origin and function of these cells in various disease models.

**TABLE 2:** Key phenotypic patterns of normal stem cells, progenitor cells, monocytes, and tissue‐resident histiocytic and dendritic cells.

Cell type	Key phenotypic features	Additionally expressed early or lineage‐related antigens^a^
Hematopoietic stem cell	CD45^+^/CD34^+^/CD38^−^	CD123 (IL‐3RA), CD117 (KIT), CD33, CD44, CD133
Hematopoietic progenitor cell	CD45^+^/CD34^+^/CD38^+^	CD123 (IL‐3RA), CD117 (KIT), CD11b, CD13, CD33, CD44
Classical monocyte	CD14^++^/CD16^−^	CD11b, CD13, CD15, CD33, CD123
Intermediate monocyte	CD14^++^/CD16^+^	CD11b, CD13, CD15, CD33, CD123
Non‐classical monocyte	CD300e^+^/CD14^+/−^/CD16^++^	CD11b, CD13, CD15, CD33, CD123
M1 macrophage	CD14^+^/CD11c^+^/CD209^−^	CD40, CD80, CD86
M2 macrophage	CD14^+^/CD11c^−^/CD209^+^	CD163, CD206
Monocyte‐related dendritic cell	CD14^+^/CD11c^+^	CD206, CD209, CD304 (BDCA4)
Macrophage‐related dendritic cell	CD14^+^/CD11b^+^	CD141 (BDCA3), CD209
Langerhans cell	CD1a^+^/EpCAM^+^/Langerin (CD207)^+^	CD11b, CD11c, CD141, E‐Cadherin
Myeloid dendritic cell type 1 (DC1)	CD141^+^/XCR1^+^	CD1a, CD11c, CLEC9A (CD5^+^ and CD5^−^ subset)
Myeloid dendritic cell type 2 (DC2)	CD1c^+^/SIRPα^+^	CD1a, CD11c, CD207
Plasmacytoid dendritic cell	CD303^+^/CD304^+^ (BDCA2^+^/BDCA4^+^) HLA‐DR^+^	CD123, CD141 (CD56^+^ subset)
Mast cells	CD34^−^/CD117^++^	CD33, CD44, IgERIA (CD88^+^ and CD88^−^ subset)

Abbreviations: BDCA, blood dendritic cell antigen; CLEC9A, C‐type lectin domain containing 9A; IgERIA, high‐affinity immunoglobulin E receptor I alpha chain.

^a^ Depending on cell type, activation state, and pathology, some surface antigens are only expressed on subsets of these cells. The table refers to normal and reactive conditions.

Regarding neoplastic states, the World Health Organization (WHO) recently updated its proposal to classify monocyte/macrophage‐related, histiocytic and dendritic cell (DC) neoplasms, and mast cell neoplasms (Table 3) [27, 28, 29, 30, 31]. A similar classification has been proposed by an international consensus classification (ICC) panel (Table S2) [31, 32]. However, although diagnosis and prognostication in these disorders improved substantially over the past decades, several questions remain concerning the optimal management of patients and the development of novel personalized treatment approaches.

**TABLE 3:** Overview of neoplasms involving tissue‐resident myeloid and histiocytic cells as defined by the World Health Classification (WHO) 2022.^a^

Acute myeloid leukemias (AML) involving the monocyte lineage
Acute myelomonocytic leukemia
Acute monocytic leukemia
Myeloid sarcoma (“monoblastic form”)
Chronic myeloid leukemias involving the monocyte lineage^b^
Juvenile myelomonocytic leukemia (JMML)
Chronic myelomonocytic leukemia (CMML)
Cutaneous mastocytosis (CM)
Maculopapular cutaneous mastocytosis (MPCM)
Diffuse cutaneous mastocytosis (DCM)
Cutaneous mastocytoma
Systemic mastocytosis (SM)
Non‐advanced systemic mastocytosis
Advanced systemic mastocytosis
Mast cell sarcoma (MCS)
Plasmacytoid dendritic cell neoplasms
Mature plasmacytoid dendritic cell proliferation associated with myeloid neoplasm
Blastic plasmacytoid dendritic cell neoplasm (BPDCN)
Langerhans cell and other (myeloid) dendritic cell neoplasms
Langerhans cell neoplasms
Other dendritic cell neoplasms
Histiocytic neoplasms
Juvenile xanthogranuloma (JXG)^c^
Erdheim‐Chester disease
Rosai‐Dorfman disease
ALK‐positive histiocytosis
Histiocytic sarcoma

^a^ These disease variants have been described in the 2022 update of the WHO classification [27]. Detailed diagnostic criteria for each variant are provided in the available literature [27, 28, 29].

^b^ In these leukemias, monocytic cells are a prominent fraction of neoplastic cells. However, absolute monocytosis is also detected in chronic myeloid leukemia (CML).

^c^ JXG is most often localized in the skin and may be caused by mutations in tissue‐resident macrophages.

## 2 Origin and Phenotype of Tissue‐Resident Myeloid Cells

Most tissue‐resident myeloid cells are considered to be derived from CD34+ multipotent or lineage‐restricted hematopoietic stem and/or progenitor cells (Table 2) [1, 2, 3, 4, 5, 6, 19, 20, 21, 22, 23, 24, 33, 34, 35]. Whereas some tissue‐fixed myeloid cells are directly replenished from a local pool of CD34+ stem and progenitor cells, other local myeloid cells develop from circulating (tissue‐entering) monocytes or from monocyte‐derived macrophages (Figure 1) [4, 5, 6, 11, 19, 23, 24, 25]. The local pools of CD34+ stem and progenitor cells may either be replenished from circulating, BM‐derived stem cells or local organ‐specific stem cells in extramedullary organs (Figure 1, Table 2). As mentioned before, some tissue‐resident myeloid cells may originate from yolk‐sac progenitors [22, 23, 24, 25, 26].

Most normal (non‐clonal/non‐neoplastic) hematopoietic stem cells giving rise to myeloid cells reside in a CD34+/CD38‐ fraction [33, 34, 35]. By contrast, normal CD34+ progenitor cells (more mature compared to stem cells) also display CD38 as well as myeloid differentiation antigens. Normal hematopoietic stem and progenitor cells typically co‐express CD33, CD44, CD117 (KIT) and low amounts of IL‐3 receptor alpha chain CD123 (Table 2) [36, 37, 38]. In peripheral tissues, such stem cells give rise to mature myeloid cells, including histiocytic cells and mast cells, depending on the organ system [1, 2, 3, 24, 25, 26, 39, 40, 41, 42, 43, 44]. Lineage commitment and maturation of these cells are also determined by local stem cell niches in the BM and extramedullary organs, including lymphatic organs (lymph nodes, spleen), skin, lung, or the gastrointestinal (GI) tract [24, 25, 26, 39, 40, 41, 42, 43, 44, 45].

Another important aspect is the varying time of development of tissue‐resident myeloid cells. Based on cell culture studies and in vivo models, differentiation and maturation of certain myeloid cells (e.g., macrophages or mast cells) from their BM‐derived or circulating stem and progenitor cells may take several weeks to months [1, 2, 3, 42, 43, 44, 45, 46]. Moreover, the lifetime of these cells is sometimes excessive, ranging from several months to years [1, 2, 3, 4, 5, 6, 42, 43, 44, 45, 46, 47, 48].

In neoplastic states, the situation is similar [19, 20, 21, 22]. Here, neoplastic myeloid cells derive from one or more subclone‐specific neoplastic stem cells (NSC), also known as leukemic stem cells (LSC) in leukemia contexts [49, 50, 51, 52, 53, 54, 55, 56]. In advanced neoplasms, such as acute myeloid leukemia (AML), the time of development of leukemic cells from their LSC is often short, both in patients and when these cells are transplanted into immunodeficient mice [51, 52, 53, 54, 55, 56].

The origin of myeloid neoplasms from disease‐propagating stem cells has obvious implications for designing curative (LSC‐eliminating) therapies [51, 52, 53, 54, 55, 56]. For example, some LSC markers and phenotypes are disease‐specific and thus diagnostic. In addition to CD34 and other classical stem cell markers, LSC displays several disease‐related or lineage‐associated surface antigens (Table 2) [38, 43, 54, 56]. It is also worth noting that LSC eradication is often followed by long‐term disease‐free survival and that allogeneic hematopoietic stem cell transplantation (HSCT) sometimes even works as an LSC‐eradicating (curative) approach in drug‐resistant patients [22, 54, 55, 56, 57, 58, 59, 60].

After lineage commitment, all maturing cells lose CD34 and start to express lineage‐related antigens and lineage‐specific antigen profiles. Surface markers typically expressed in various lineages are shown in Table 2. Several surface antigens are also associated with a specific function, such as adhesion, migration, or activation, or serve as therapeutic targets in neoplastic states.

The role and impact of tissue‐resident leukocytes in inflammatory reactions, atherosclerosis, other vascular disorders, and solid tumors is described in the supplement of this manuscript. Neoplasms originating from monocytic cells, histiocytic cells, mast cells, and their stem and progenitor cells are reviewed and discussed in the following paragraphs.

## 3 Monocytic and Myelomonocytic Leukemias

Mature and immature monocytes are usually part of the malignant clone in diverse myeloid neoplasms, including myelodysplastic syndromes/neoplasms (MDS), myeloproliferative neoplasms (MPN) and myeloid leukemias. Chronic leukemias typically presenting with monocytosis include chronic myeloid leukemia (CML) where only absolute monocytosis is found, juvenile myelomonocytic leukemia (JMML) and chronic myelomonocytic leukemia (CMML), where relative and absolute monocytosis are usually present (Table 3) [27, 32, 61, 62, 63, 64, 65]. Clonal monocytes in CMML and other chronic myeloid neoplasms are CD14+ cells. In myelomonocytic and monocytic AML, clonal monocytic cells may also be detected. A morphologic classification of monocytic cells and their progenitors detectable in myeloid leukemias, together with morphologic criteria, is shown in Table S5 [62].

Patients with JMML usually present with splenomegaly, leukocytosis and monocytosis, and a hypercellular BM. Blast cells may be increased but are less than 20% [27, 32, 61, 62]. CMML is usually diagnosed in adults and defined by relative and absolute monocytosis [27]. In most cases, leukocytosis and splenomegaly are detected [27, 62]. Depending on the blast count, CMML is split into CMML‐1 (BM blasts < 10% and PB blasts < 5%) and CMML‐2 (BM blasts 10%–19%, circulating blasts 5%–19%). In both JMML and CMML, the presence of AML and CML must be excluded by definition [27, 32]. However, both types of neoplasms can transform into overt (secondary) AML [27, 62].

Based on the 2022‐updated WHO classification (5th Edition), many AML variants are defined by molecular and cytogenetic aberrations [27]. However, two types of AML are defined by monocytic differentiation, namely acute myelomonocytic leukemia (myelomonocytic AML) and acute monocytic leukemia (monocytic AML) [27]. For myelomonocytic AML, the following criteria apply: (i) at least 20% of all cells are monocytes or monocyte precursors, (ii) ≥ 20% of cells are maturing granulocytic cells, and (iii) ≥ 3% of the blasts are positive for myeloperoxidase (MPO) [27]. Monocytic AML is defined by the following criteria: (i) ≥ 80% of cells are monocytes and/or monocyte precursors, (ii) < 20% of cells are maturing granulocytes, and iii) blasts and promonocytes (monocytic cells) must express at least two of the following markers: CD11c, CD14, CD36, or CD64 or show non‐specific esterase (NSE) positivity [27].

Other previously recognized myelomonocytic and monoblastic variants of AML (previous WHO proposals) are now defined by specific molecular and/or cytogenetic lesions. For example, patients previously diagnosed as “acute myelomonocytic leukemia with eosinophilia” (AML‐M4‐eo) are now defined by the presence of the *CBFB::MYH11* fusion‐product and the related karyotype anomalies inv(16)(p13.1q22) or t(16;16)(p13.1q22) [27]. Likewise, monoblastic AML carrying mutations in *NPM1* are now defined as *NPM1*‐mutated AML.

The ICC proposal follows a similar concept [32]. However, no defined monocytic AML variants are included in the ICC. Moreover, the ICC recognizes not only CMML, but also two potential pre‐CMML stages, namely (i) clonal cytopenia with monocytosis of undetermined significance and ii) clonal monocytosis of undetermined significance (Table S2) [32].

The prognosis of CMML, monocytic AML, and other AML variants with monocytic involvement depends on age, the type and number of molecular and cytogenetic lesions, and response to remission‐induction therapy [62, 63, 64, 65, 66, 67, 68]. In general, the prognosis is poor in advanced CMML, RAS‐driven CMML, *NPM1*‐mutated CMML (recognized by ICC), and drug‐resistant CMML [62, 63, 64, 65, 66, 67, 68]. In those where such a disease precedes the development of AML, the AML is regarded as “secondary” (sAML). In many of these cases, chemotherapy followed by allogeneic HSCT is recommended [63, 64, 65]. An advanced histiocytic or mast cell neoplasm may also be indicative of a poor prognosis [21, 62, 68]. However, a concomitant indolent systemic mastocytosis, indolent histiocytic neoplasm, or a small‐sized *JAK2*‐mutated MPN clone may not necessarily influence the prognosis of patients with CMML [69, 70, 71].

## 4 Mastocytosis and Associated Myeloid Neoplasms

In the recent past, several novel concepts and approaches in the field of systemic mastocytosis (SM) have been developed. Some of these patients develop an associated hematologic neoplasm (AHN). The diagnosis of SM is based on major and minor diagnostic SM criteria (Table S6) [22, 28, 29, 72, 73, 74]. When at least 1 major and 1 minor or at least 3 minor SM criteria are fulfilled, the diagnosis of SM can be established (Table S6) [28, 29, 72, 73, 74]. The cells of origin in SM are multipotent and/or mast cell‐committed stem and progenitor cells [22, 75]. Neoplastic mast cells are replenished from these cells and exhibit a characteristic phenotype, including pan‐leukocyte and/or pan‐mast cell markers (CD33, CD44, CD45, CD117/KIT, tryptase) and aberrant surface markers, namely CD2 (LFA‐2), CD25 (IL‐2RA), and CD30 (Ki1‐antigen) [76, 77, 78, 79]. Aberrant expression of CD2, CD25 and/or CD30 also serves as a minor diagnostic criterion of SM (Table S6) [72, 73, 74].

Based on WHO criteria, mastocytosis is divided into cutaneous mastocytosis (CM), SM, and mast cell sarcoma (MCS) (Table S7) [27, 28, 29, 72, 73, 74]. Whereas patients with CM and those with non‐advanced forms of SM have an excellent prognosis concerning progression and survival, patients with advanced SM, including aggressive SM (ASM), SM with an AHN (SM‐AHN) and mast cell leukemia (MCL) have a grave prognosis (Table S7) [80, 81, 82, 83]. In patients with SM‐AHN, the AHN are mostly myeloid neoplasms which has clinical implications and prompted the ICC group to propose the term “associated myeloid neoplasm (AMN)” [32]. Patients with SM‐AHN/AMN may present with an MDS, MPN, MDS/MPN overlap‐disease, or AML [84, 85, 86, 87]. The most prevalent AHN/AMN‐forms are CMML and AML [84, 85, 86, 87]. Among SM‐AHN, lymphoid AHN are very rare and no longer recognized in the ICC [32]. In a recent harmonization‐proposal of the EU/US consensus group, lymphoid AHN are again recognized and termed associated lymphoid neoplasms (ALN) [31]. In very rare cases, SM may also co‐exist with a histiocytic or DC neoplasm, such as a blastic plasmacytoid DC neoplasm (BPDCN) (K. Sotlar, H.P. Horny, P. Valent, unpublished observation).

In most patients with SM, the KIT‐activating point mutation D816V is detected and is regarded as a major disease driver [72, 73, 74, 86, 87, 88]. In myeloid AHN, *KIT* D816V may also be detected, especially in CMML (> 80% cases) and AML (50%–70% of all cases) [86, 87, 88, 89]. In advanced SM, neoplastic cells (mast cells and AHN cells) often express mutations in additional critical genes, such as *ASXL1*, *TET2*, *RUNX1*, or *SRSF2*. An important aspect is that *KIT* D816V is usually not expressed in lymphoid AHN cells [72, 73, 74, 90]. Whether *KIT* D816V is expressed in BPDCN cells in patients with SM‐BPDCN remains unknown.

SM is considered a stem cell neoplasm derived from multi‐potent or lineage‐restricted neoplastic stem cells (LSC) [75, 91]. Whereas survival and the quality of life (QOL) were poor in advanced SM until recently, the advent of new potent KIT D816V‐targeting drugs (midostaurin, avapritinib, others) and other potent therapies, including HSCT, has greatly improved progression‐free and overall survival in these patients [74, 83, 92].

Finally, it is important to note that patients with SM may also suffer from severe mediator‐induced symptoms (e.g., anaphylaxis) [17, 93, 94, 95, 96, 97]. Especially in patients with concomitant IgE‐dependent allergies such as *Hymenoptera* venom allergy and/or hereditary alpha tryptasemia (HαT), severe life‐threatening anaphylaxis and a mast cell activation syndrome (MCAS) may be diagnosed [22, 93, 94, 95, 96, 97, 98]. In these patients, anti‐mediator‐type drugs (histamine receptor blocker), emergency medication (epinephrine, glucocorticosteroids), allergen‐specific immunotherapies (for bee/wasp venom allergy) or anti‐IgE‐antibody therapy have improved outcomes and survival [93, 94, 95, 96, 97, 98, 99]. In addition, novel KIT‐targeting drugs, like avapritinib, have recently been shown to reduce the mast cell burden and mast cell activation in these patients [100, 101].

## 5 Histiocytic and Dendritic Cell Disorders

Histiocytic and DC disorders include a variety of conditions and pathologies defined by abnormal growth and accumulation of tissue‐resident histiocytic cells [18, 19, 20, 21, 23, 24]. Histiocytic and DC neoplasms can essentially be divided into non‐aggressive and aggressive/malignant forms [18, 19, 20, 21]. In addition, histiocytic disorders can be classified based on the organ(s) and cell types involved [18, 19, 20, 21].

Table S8 shows a clinical classification of histiocytic disorders proposed by a Histiocyte Society consensus group [20]. In this proposal, these conditions are divided into (i) a cutaneous “C” group, (ii) a hemophagocytic “H” group (including hemophagocytic lymphohistiocytosis, HLH), (iii) a Langerhans cell “L” group (including Langerhans cell histiocytosis and Erdheim‐Chester disease), iv) a malignant myeloid “M” group (including malignant forms defined by the WHO), and v) a Rosaí Dorfman “R” group (including Rosaí Dorfman disease = RDD) [20].

The WHO classification of histiocytic and DC neoplasms is shown in Table 4 [27, 102]. The WHO describes two forms of plasmacytoid DC neoplasms, namely a “(mature) plasmacytoid DC proliferation associated with myeloid neoplasm” where the associated myeloid neoplasm (CMML, MDS, and AML) is advanced and prominent and often driven by “RAS‐pathway mutations,” and BPDCN where DC are more immature and DC accumulation is prominent initially while an overt myeloid neoplasm (CMML, AML) may or may not develop later during follow up (Table 4) [27, 102]. In some patients, the concomitant myeloid neoplasm (CMML) may precede the BPDCN.

**TABLE 4:** Histiocytic and dendritic cell neoplasms defined by the WHO.

Neoplasm and categories/subsets	Proposed cell of origin
1. Plasmacytoid dendritic cell neoplasms	Plasmacytoid dendritic cell (pDC: CD56+/CD123+) and/or their stem/progenitor cells (CD34+/CD117+/CD123+)
1.a. (Mature) Plasmacytoid dendritic cell proliferation associated with myeloid neoplasm^a^	Hematopoietic stem and/or progenitor cells giving rise to dendritic cell neoplasm and to an associated myeloid neoplasm*
1.b. Blastic plasmacytoid dendritic cell neoplasm (BPDCN)	Plasmacytoid dendritic cell (and/or lineage‐restricted progenitor cells)
2. Langerhans cell and other (myeloid) dendritic cell neoplasms	Dendritic cells (CD45+/CD68+)
2.a. Langerhans cell neoplasms	Langerhans cells:
‐ Langerhans cell histiocytosis (LCH)	CD1a+/CD207+
‐ Langerhans cell sarcoma	CD1a+/CD207+/S100+
2.b. Other dendritic cell neoplasms	Myeloid dendritic cells:
‐ Indeterminate dendritic cell tumor	CD1a+/CD1c+/CD207‐
‐ Interdigitating dendritic cell sarcoma	S100+/CD1a‐/CD207‐
3. (Other) Histiocytic neoplasms	Histiocytic myeloid cells and/or their progenitors:
‐ Juvenile xanthogranuloma	CD68^+^/CD163^+^ histiocytes
‐ Erdheim‐Chester disease	CD68^+^/CD163^+^ histiocytes
‐ Rosai‐Dorfman disease	CD68^+^/CD163^+^ histiocytes
‐ ALK‐positive histiocytosis	ALK^+^ histiocytes
‐ Histiocytic sarcoma	CD68^+^/CD163^+^ histiocytes

Abbreviations: ALK, anaplastic lymphoma kinase; WHO, World Health Organization.

^a^ These disease variants have been described in the 2022 update of the WHO classification [27].

The second WHO group is called “Langerhans cell and other DC neoplasms” and includes “Langerhans cell histiocytosis” (LCH) and “Langerhans cell sarcoma” (Table 4) [27]. In addition, this group includes “indeterminate dendritic cell tumor” and “interdigitating dendritic cell sarcoma” [27, 103]. The third group is called “histiocytic neoplasms” and includes “juvenile xanthogranuloma,” “Erdheim‐Chester disease,” “Rosai‐Dorfman disease,” “ALK‐positive histiocytosis,” and “histiocytic sarcoma “(Table 4) [27, 102]. In some of the histiocytic neoplasms, especially in Langerhans cell histiocytosis and Erdheim‐Chester disease, neoplastic cells often display mutations in genes triggering the MAPK pathway, including mutations in *BRAF* (most prevalent: V600E), *ARAF*, *MAP2K1*, *NRAS* or *KRAS* [27]. It is also worth noting that ALK‐positive histiocytosis is the first molecularly defined histiocytic neoplasm, as it is characterized by *ALK* rearrangements, such as the *KIF5B::ALK* fusion.

A similar classification has been proposed by the ICC (Table S9) [32, 104]. However, whereas most of the histiocytic and DC neoplasms are listed among lymphoid and histiocytic cell and DC neoplasms, BPDCN is listed among myeloid neoplasms [32, 104]. In addition, the ICC contains two additional (new) variants of neoplasms: (i) the so‐called “fibroblastic reticular cell sarcoma” and (ii) Epstein–Barr virus–positive inflammatory follicular dendritic cell/fibroblastic reticular cell tumor (Table S9) [32, 104].

In the following paragraphs, we provide clinical and genetic features and phenotypes in histiocytic and DC disorders.

Langerhans Cell Histiocytosis (LCH) is found in children and young adults and may affect any organ system, including the skin, bones (mastoid lesions and vertebral bone damage), BM (cytopenia), the GI tract (chronic diarrhea and weight loss), liver and spleen (hepatosplenomegaly), and the central nervous system (CNS). Typical CNS findings include a pituitary gland mass leading to diabetes insipidus, cerebellar dysfunction, and a neurodegenerative disease [18, 19, 20, 21]. When assessed histologically, LCH lesions contain neoplastic CD1a+, CD207+ Langerhans cell‐like cells [18, 19, 20, 21]. In most patients, LCH sites also show signs of inflammation. Some of these patients may even develop massive hyper‐inflammation resembling secondary HLH [20]. In about half of the patients, the *BRAF* mutation V600E is detected [18, 19, 20, 21]. In other cases, mutations in genes of the MAPK pathway, including *MAP2K1*, *ARAF*, *NRAS*, or *KRAS*, or other *BRAF* mutations, are found [18, 19, 20, 21]. LCH may progress and disseminate into multiple organs and show a more or less rapidly progressive clinical course [18, 19, 20, 21]. In these patients, systemic chemotherapy (e.g., vinblastine and prednisone) is often recommended as a first‐line approach. Moreover, targeted therapy with BRAF blocker (e.g., vemurafenib and dabrafenib) or MEK inhibitors (e.g., cobimetinib and trametinib) has shown promising results in refractory LCH [103, 105, 106]. In other patients, a stable disease is recorded, and in very few cases (with early lesions) spontaneous regression is seen.

Erdheim Chester Disease (ECD) occurs primarily in adults and can also affect one or multiple organs (most frequently long bones, kidney, skin, brain, lung, heart) and can also show a stable or progressive disease course [18, 19, 20, 21]. However, unlike LCH, no spontaneous regression has been reported. Some patients may also develop CNS involvement (“tumorous” or “pseudo‐degenerative”) and many are diagnosed because of diabetes insipidus (detected sometimes many years before ECD is diagnosed) [18, 19, 20, 21]. Other typical findings include symmetric sclerosis of long bones, “hairy kidney,” “coated aorta,” right atrial masses (pseudo‐tumor) and/or xanthelasma [18, 19, 20, 21]. ECD cells are macrophage‐like (foamy, lipid‐laden) histiocytes that display CD14, CD68, and CD163, and stain negative for CD1a and CD207 [18, 20]. Like in LCH, the *BRAF* V600E mutation is detected in about half of the patients [18, 19, 20, 21]. In the remaining cases, mutations in other genes related to the RAS–RAF‐MAP pathway may be found [18, 20].

Rosai Dorfman Destombes disease (RDD), also known as “sinus histiocytosis with massive lymphadenopathy” is an ill‐defined and clinically heterogeneous disease that is characterized by histiocytic infiltration of the lymph nodes, skin, upper respiratory tract, eye and retro‐orbital tissue, and less frequently bones, kidney, or parotid gland [18, 19, 20, 21]. RDD cells express classical macrophage markers (CD14, CD68, and CD163) as well as S100 and OCT2, but lack LCH‐related antigens [20]. In a subset of RDD patients, mutations in genes fueling the RAS–RAF‐MAP kinase pathway are found [18, 19, 20]. The prognosis of RDD patients is favorable compared to LCH or ECD. In many cases, no therapy is required. In other patients, targeted therapy (e.g., MEK inhibitors), surgery (resection of enlarged lymph nodes) and/or chemotherapy are recommended.

Histiocytic sarcoma (HS) is an extremely rare hematopoietic neoplasm that originates from the macrophage lineage and shows a sarcoma‐like spread of CD4+, CD68+, PU.1+, and CD163+ histiocytic cells [20, 27, 107, 108, 109]. In a subset of patients, neoplastic cells display activating mutations in *BRAF* (including V600E and others), *KRAS*, *NRAS*, and *MAP2K1* and *KMT2D* [20, 107]. HS can affect one or multiple organ systems, including the skin, superficial and deep soft tissue, lung, nasal cavity, GI tract, or spleen [20, 27, 107, 108, 109]. CNS or lymph nodes may also be affected. HS may be diagnosed as an isolated disease or in association with another hematologic neoplasm, such as a B‐cell lymphoma, multiple myeloma, MDS, or acute leukemia [20, 107, 108, 109]. In most patients with rapid dissemination, the clinical condition deteriorates rapidly despite therapy, and most patients succumb to their disease within 1 year [20, 107, 108, 109]. Approximately a third of the cases are clonally related to a co‐existent B‐cell lymphoma, with clonal Ig‐rearrangement and lymphoma‐related molecular alterations. However, when detected early and treated with intensive therapy, some patients with localized HS may survive for a longer time.

BPDCN is a rare hematologic malignancy that involves certain pDC subsets or their precursor cells (pre‐pDC) and sometimes even pluripotent stem cells. The disease usually shows an aggressive clinical course and is associated with a poor prognosis [20, 21, 27]. BPDCN is often discovered (suspected) due to its typical, mostly bruise‐like, skin lesions. However, BPDCN usually involves multiple organ systems, including the BM (causing cytopenia), and less frequently lymph nodes, spleen, peripheral blood, and/or CNS [110, 111, 112, 113]. In a subset of patients (20%–30%), a myeloid neoplasm, such as a CMML, MDS, or AML, is also present or precedes BPDCN [110, 111, 112, 113]. When clonal pDC are detected in the context of a predominant AML, the term pDC‐AML is used. While BPDCN includes also immature pDC forms, mature pDC proliferations (low Ki67 expression and loss of CD56) may also be found, often in CMML contexts. This condition is called mature pDC proliferation (MPDCP) [110, 111, 112, 113]. Immature pDC are blast‐like cells that typically express CD117 and CD123^hi^ and sometimes also CD34 [110]. While the WHO describes both BPDCN and MPDCP, the ICC only lists BPDCN [27, 32].

According to the WHO classification, the diagnosis of BPDCN can be established when neoplastic cells (i) express CD123* and either CD4 or CD56 (or both) as well as 1 of the following: TCF4*, TCL1*, CD303*, CD304* or (ii) express ≥ 3 of the above‐mentioned DC markers (marked by asterisk: *) but lack all of the following: CD3, CD14, CD19, CD34, lysozyme, and myeloperoxidase (Table 5) [27]. This means that BPDCN cells can co‐express CD34 (very immature pDC) when “(i)” is fulfilled.

**TABLE 5:** Diagnostic phenotypic criteria of blastic plasmacytoid dendritic cell neoplasm (BPDCN) as defined by the WHO.

A: Markers expected to be expressed in neoplastic pDC in BPDCN: CD123*, TCF4*, TCL1*, CD303*, CD304*, CD4, CD56
B: Markers expected to stain negative in PBDCN cells: CD3, CD14, CD19, CD34, Lysozyme, Myeloperoxidase
→
Minimal diagnostic criteria of PBDCN defined by phenotyping^a^:
1) Expression of CD123 and one additional pDC marker (*) in addition to CD4 and/or CD56 (at least one of these 2 antigens)
or/and
2) Expression of at least three pDC markers (any marker listed in “A”) and absent expression of all expected negative markers (listed in “B”).

Abbreviations: pDC, plasmacytoid dendritic; WHO, World Health Organization.

^a^ The diagnosis PBDCN can be established when: (1) is fulfilled or/and (2) is fulfilled.

Molecular abnormalities include mutations in *TET2*, *ASXL1*, *RAS*, and other genes, most of which can also be found in associated myeloid neoplasm(s) [111, 112, 113]. The prognosis of patients with BPDCN and MPDCP is poor. In many cases, the course is dictated by an associated myeloid neoplasm (e.g., CMML or AML) [111, 112, 113]. The outcome and survival in BPDCN and MPDCP patients also depend on responses to induction therapy. In well‐responding patients who are eligible and have a suitable donor, HSCT is often recommended.

Hemophagocytic lymphohistiocytosis (HLH) is a clinical syndrome that is characterized by massive hyper‐inflammation with a cytokine storm and fever, hemophagocytosis in the BM and other lympho‐hematopoietic organs, and a poor prognosis [20, 114]. In many patients, HLH is rapidly progressing [20, 114]. Although idiopathic variants have been proposed, many patients have an underlying disease process, thus designated secondary HLH, or a genetic predisposition [114]. Underlying disorders include hematologic neoplasms such as myeloid, histiocytic, or lymphatic disorders, solid tumors, and non‐malignant conditions with overwhelming inflammatory reactions of the immune system (examples: M. Still or VEXAS syndrome) [20, 114]. Other patients have an inherited disorder predisposing to HLH, and are classified as (i) familial HLH (due to mutations in *PRF1*, *UNC13D*, *STX11*, or *STXBP2*), (ii) pigmentary disorders associated with HLH, due to mutations in *RA827A* (Griscelli syndrome type 2), *LYST* (Chediak‐Higashi syndrome) or *AP3B1* (Hermansky‐Pudlak syndrome 2), (iii) X‐linked lymphoproliferative diseases (XLP) due to mutations in *SH2D1A* (XLP1) or *XIAP* (XLP2), and iv.) EBV susceptibility disorders (several mutations in target genes that can disturb immune cell defense) [20]. These underlying (predisposing) conditions and disorders have to be delineated distinctively from the HLH syndrome itself where specific diagnostic clinical criteria have to be met (Table S10) [20]. These criteria include (i) fever, (ii) splenomegaly, (iii) marked cytopenia in at least two cell lineages, (iv) hypertriglyceridemia (> 265 mg/dL) or hypofibrinogenemia (< 150 mg/dL), (v) hyperferritinemia (>500 ng/mL; in most cases much higher levels are found), vi.) elevated serum levels of soluble CD25, and (vii) hemophagocytosis in BM, spleen, lymph nodes, or liver [20, 114, 115, 116, 117]. Based on the proposal of the Histiocyte Society, the diagnosis HLH can be established when 5 out of these 7 criteria are fulfilled (Table S10) [20, 114, 115, 116, 117]. Although these criteria were initially developed to support the conduct of clinical trials, we recommend that the same criteria are also used in daily practice. It is also important to note that in adults, secondary HLH, triggered by hematologic disorders, is the leading HLH subtype [20, 114, 115, 116, 117]. For these patients, adapted threshold levels for ferritin (1000 ng/mL) and soluble CD25 (3900 U/mL) have been demonstrated to optimize HLH diagnosis [114]. Therefore, we recommend integrating such optimized thresholds in clinical practice, for example in those with hematologic neoplasms or fever of unknown origin, and to use these adapted parameters in combination with the modified HLH‐2004 criteria [114, 115, 116, 117].

## 6 Novel Developments in Diagnosis and Prognostication

From a clinical point of view, one of the most important aspects is that the clinician must delineate distinctively between (i) underlying disease processes defined by histopathological, morphological, immunological, and molecular features/criteria (e.g., histiocytosis, mastocytosis, or CMML), and (ii) various clinical syndromes that are produced by underlying disorders and are defined by distinct clinical criteria. Underlying conditions may be clonal neoplasms but may also be reactive states, and both may (or may not) produce an identical clinical syndrome. In the field of histiocytic disorders, the paradigmatic example of such a syndrome is HLH [20, 114], and in the field of mast cell disorders, the key example is MCAS [93, 94, 95]. An overview of these clinical syndromes (digest) is shown in Table S11. It is important to note that in each case, both i) the underlying disease and ii) the syndrome must be diagnosed using generally accepted (minimal) diagnostic criteria, and that both conditions have to be managed with specific therapy. It is also worth noting that some of the syndromes, for example tumor‐lysis syndrome, may be provoked by (antineoplastic) therapy (Table 11).

During the past 5 years, novel tools and technologies have revolutionized diagnostic approaches and prognostication in applied hematology, including rare hematologic neoplasms and early clonal conditions preceding various blood cancer types. These technologies include, among others, computer‐based high‐capacity (single cell) screens to detect abnormal morphologies of blood and BM cells by computed‐based microscopy, or large‐scale multi‐color flow cytometry techniques to improve minimal residual disease (MRD) diagnostics [118, 119, 120, 121, 122]. In addition, new highly sensitive PCR screens and whole exome/genome sequencing techniques have been developed to identify early clonal stages of various blood cell disorders and to quantify MRD in patients with blood cell disorders during “potentially curative” treatments [120, 121, 122]. Several of these approaches are now supported by robot‐based algorithms and artificial intelligence (AI) [120, 121, 122]. Such AI‐supported approaches are largely restricted to highly specialized laboratories, but are expected to be used more broadly in various routine laboratories within the next few years.

In the recent past, a number of prognostic scoring systems used in various groups of patients with monocytic, histiocytic, or mast cell neoplasms have been established [80, 81, 123, 124, 125]. These scoring systems have been developed on the basis of larger studies including several thousand cases collected even in rare disorders in larger international multi‐center registries. For example, the registry of the European Competence Network in Mastocytosis (ECNM) has collected over 7000 patients in a rare disease between 2012 and 2025 [81]. Similar networks have also been developed in the fields of histiocytosis (LCH), MDS, AML, and CMML [123, 124, 125, 126, 127, 128].

## 7 Novel Emerging Treatment Concepts

In the past few decades, a number of new effective targeted drug therapies, immunotherapies, and cell therapies have been developed. In several myeloid neoplasms, HSCT is currently still the only curative approach, such as in monocytic leukemias (e.g., CMML or AML), advanced SM, and malignant forms of histiocytic and DC neoplasms [60, 92, 129, 130, 131, 132, 133, 134, 135, 136]. Many current therapeutic algorithms focus on the optimal preparation for HSCT of eligible patients, especially when these cases have drug‐resistant disease [60, 129, 130, 131, 132, 133, 134, 135, 136]. One approach is to apply cytotoxic antibodies or antibody constructs directed against more or less specific cell surface antigens or checkpoint molecules, with the advantage that antibody‐based therapies usually work independent of oncogenic signaling pathways in neoplastic cells. With such drugs, several patients can be de‐bulked and bridged to HSCT. Highlighting examples are gemtuzumab‐ozogamicin (CD33‐directed toxin‐conjugate) in AML or tagraxofusp (CD123‐directed toxin‐conjugate) in patients with BPDCN [137, 138, 139, 140].

Another strategy is to target the primary oncogenic lesion (oncoprotein), such as KIT D816V in advanced SM or BRAF V600E in advanced forms of histiocytic neoplasms [74, 83, 103, 105, 106, 141, 142, 143, 144]. Even if no *BRAF* mutation is found, other RAS‐pathway mutations are often detected, and the RAS–RAF‐MAP‐kinase pathway may play a role in disease progression. Such RAS‐pathway driven neoplasms encompass advanced monocytic and histiocytic neoplasms as well as RAS‐driven AML. Therefore, inhibitors of this pathway are currently being tested in clinical trials. In patients with ALK‐positive histiocytosis, ALK inhibitors have recently been tested and shown to exert impressive anti‐neoplastic effects [145]. In advanced SM, the novel KIT D816V‐targeting drug avapritinib induces major clinical responses and even complete hematologic remission in a high proportion of cases [141, 142]. Compared to previous (best available) therapies, avapritinib and similarly strong KIT inhibitors are considered game‐changers in the treatment landscape of SM [142]. However, in many patients with advanced SM or histiocytic disease, an associated MDS, CMML, or AML is also present, which requires additional therapeutic considerations. Moreover, an additional clinical syndrome such as MCAS or HLH may be diagnosed. These associated conditions and pathologies can only be managed in an optimal way when personalized medicine approaches are applied [146, 147, 148]. For example, more and more specific targets and target pathways have been identified, and many potent and specific drugs have been developed. These precision medicine tools and drugs can now be tested in clinical trials in defined patient subsets, which will result in a new era of medicine, best described as personalized precision medicine. This new era is of major importance, especially in rare diseases, including neoplasms involving tissue‐resident myeloid cells. This new field of personalized medicine will further improve survival and quality of life in such patients. We also conclude that basic and translational research is important to establish new therapeutic concepts in the field of histiocytic cells in the future [142, 143, 144, 145, 146, 147, 148, 149].

## Author Contributions

All authors contributed equally by screening and discussing the available literature and unpublished data and/or by discussing case reports. All authors contributed substantially by writing and reviewing parts of the manuscript. All authors approved the final version of the document.

## Ethics Statement

The authors have nothing to report.

## Conflicts of Interest

Peter Valent: 1. Research grant: AOP Orphan, 2. Advisory board and other consultancy honoraria: Novartis, Blueprint Medicines, Cogent, Pfizer, AOP Orphan, Stemline, Abbvie, Delbert, Pentabase, Daiichi‐Sankyo, Amgen; Johann Wojta: no COI; Petri T. Kovanen: Advisory Board and Honoraria: Aegerion, Amgen, Novartis, Raisio, Sanofi/Regeneron; Olivier Hermine: Research grants: Blueprint Medicines, Abbvie, Novartis, 2. Advisory Board and Honoraria: AB Science; Falko Fend: Research grants: Stemline, Thermo Fisher, 2. Advisory Board and Honoraria: Stemline, Astra Zeneca, aetherAI, Roche, EUSAPharma; Karl Sotlar: Karl Sotlar: Advisory Board and Honoraria: Novartis, Blueprint Medicines, Astra Zeneca; Hildegard Greinix: Advisory Board and Consultancy Honoraria: Novartis, Stemline, Gilead, Sanofi, Therakos, Roche, Takeda; Klaus Geissler: 1. Research grants: Otsuka, 2. Advisory Board and Honoraria: Otsuka, Beigene; Karin Hartmann: Advisory board and consultant honoraria: ALK‐Abello, Allergopharma, Almirall, BioCryst, Blueprint, Cogent, Galderma, KalVista, Leo, Menarini, Novartis, Pfizer, Sanofi, Takeda, and Thermo Fisher; Juliana Schwaab: 1. Advisory Board: Blueprint Medicines, GSK; 2. Honoraria: Novartis, GSK, Astra Zeneca, AOP Orphan; Marco Herling: 1. Research funding: Beigene and JanPix; 2. Consulting/advisory role for and honoraria from: BeiGene, Janssen, Stemline Menarini, Takeda; honoraria also from Roche; expert testimony for Stemline Menarini; 3. Travel/accommodations and expenses from BeiGene and Janssen; Laura Boccuni: no COI; Lukas Kazianka: 1. DOCmed fellowship (Austrian Academy of Sciences), 2. Honoraria: Roche, Beigene, Abbvie; Max John: no COI; Wolfgang R. Sperr: 1. Consultancy and Honoraria: Thermo Fisher, AbbVie, Novartis, Pfizer, BMS/Celgene, Blueprint, Incyte, Stemline, Servier, Jazz, Teva, 2. Research Grant: Pfizer; Carina Zierfuss: no COI; Alexandar Tzankov: Consultancy and Honoraria: Blueprint Medicines; Christian Sillaber: Consultancy and Honoraria: Takeda, Vertex; Milen Minkov: no COI; Gregor Hoermann: Advisory Board and Honoraria: AOP Orphan Pharmaceuticals, AstraZeneca, Blueprint Medicines, Cogent Biosciences, Gilead, Janssen‐Cilag Pharma, Jazz Pharmaceuticals, MSD Sharp & Dohme; Matthew Collin: no COI; Hans‐Peter Horny: Advisory Board and Honoraria: Novartis, Blueprint Medicines; Torsten Haferlach: Part owner of MLL Munich Leukemia Laboratory; Maria Sibilia: no COI; Julien Haroche: no COI; Paul La Rosée: Advisory Board and Honoraria: Johnson & Johnson, Astra Zeneca, Novartis, BMS, Sanofi; Alberto Orfao: 1. Research grants: Blueprint Medicines, ImmunosStep, 2. Advisory Board and Honoraria: Blueprint Medicines; Michel Arock: 1. Research Grant: Blueprint Medicines, 2. Advisory Board and Honoraria: AB Science, Blueprint Medicines, Novartis, Thermo Fisher. The authors declare that they have no other conflicts of interest to disclose.

## Supporting information


**Data S1:** Supporting Information.

## Data Availability

The data that support the findings of this study are available from the corresponding author upon reasonable request.
