# Supplementary material for: Tissue‐Resident Myeloid and Histiocytic Cells in Health and Disease: Novel Emerging Concepts
Source: Am J Hematol. 2025 Sep 12;100(12):2305–19. doi: 10.1002/ajh.70062 (PMC12603893; doi:10.1002/ajh.70062)
Supplement: Supplementary file 1 — Data S1: Supporting Information. [file AJH-100-2305-s001.docx]

Supplementary Appendix to

**Tissue-Resident Myeloid and Histiocytic Cells in**

**Health and Disease: Novel Emerging Concepts**

Peter Valent^1,2^, Johann Wojta^3,4^, Petri T. Kovanen^5^, Olivier Hermine^6^, Falko Fend^7^, Karl Sotlar^8^, Hildegard Greinix^9^, Klaus Geissler^10^, Karin Hartmann^11,12,13^, Juliana Schwaab^14^, Marco Herling^15^, Laura Boccuni^4^, Lukas Kazianka^1^, Max Vincent John^1,2^, Wolfgang R. Sperr^1,2^, Carina Zierfuss^16^, Alexandar Tzankov^17^, Christian Sillaber^1^, Milen Minkov^10,18^, Gregor Hoermann^19^, Matthew Collin^20^, Hans-Peter Horny^21^,

Torsten Haferlach^19^, Maria Sibilia^22^, Julien Haroche^23^,

Paul La Rosée^24^, Alberto Orfao^25^, Michel Arock^26^

^1^Department of Internal Medicine I, Division of Hematology and Hemostaseology, Medical University of Vienna, Austria; ^2^Ludwig Boltzmann Institute for Hematology and Oncology, Medical University of Vienna, Austria; ^3^Department of Internal Medicine II, Division of Cardiology; Medical University of Vienna, Austria; ^4^Ludwig Boltzmann Institute for Cardiovascular Research, Medical University of Vienna, Austria; ^5^Wihuri Research Institute, Helsinki, Finland; ^6^Service d’hématologie, Imagine Institute Université de Paris, INSERM U1163, Centre national de référence des mastocytoses, Hôpital Necker, Assistance publique hôpitaux de Paris, France; ^7^Institute of Pathology and Neuropathology and Comprehensive Cancer Center, University of Tübingen, Tübingen, Germany; ^8^Institute of Pathology, University Hospital Salzburg, Paracelsus Medical University, Salzburg, Austria; ^9^Division of Hematology, Medical University of Graz, Austria; ^10^Sigmund Freud University, Faculty of Medicine, Vienna, Austria; ^11^Division of Allergy, Department of Dermatology, University Hospital Basel and University of Basel, Switzerland; ^12^Department of Clinical Research, University Hospital Basel and University of Basel, Switzerland; ^13^Department of Biomedicine, University Hospital Basel and University of Basel, Basel, Switzerland; ^14^Department of Hematology and Oncology University Hospital Mannheim, Heidelberg University, Mannheim, Germany; ^15^University Hospital Leipzig, Department of Hematology, Cell Therapy, Hemostaseology, Infectious Diseases and Cancer Center Central Germany (CCCG) Leipzig-Jena, Germany; ^16^Department of Internal Medicine I, Division of Clinical Oncology, Medical University of Vienna, Austria; ^17^Institute of Medical Genetics and Pathology, University Hospital Basel, Basel, Switzerland; ^18^Children´s Cancer Research Institute, Medical University of Vienna, Austria; ^19^MLL Munich Leukemia Laboratory, Munich, Germany; ^20^Newcastle University, Newcastle Upon Tyne, U.K.; ^21^Institute of Pathology, Ludwig-Maximilians-University, Munich, Germany; ^22^Center for Cancer Research, Comprehensive Cancer Center, Medical University of Vienna, Austria; ^23^Pitié-Salpêtrière Hospital, French National Centre for Histiocytoses, Paris, France; ^24^Department for Internal Medicine II, Schwarzwald-Baar Klinikum, Villingen-Schwenningen, Germany; ^25^Servicio Central de Citometria, Centro de Investigacion del Cancer (IBMCC; CSIC/USAL) Instituto Biosanitario de Salamanca (IBSAL), CIBERONC and Department of Medicine, University of Salamanca, Spain; ^26^CEREMAST, Department of Hematological Biology, Pitié-Salpêtrière Hospital, Pierre et Marie Curie University (UPMC), Paris, France

**Development of Expert Statements**

The expert statements provided in this article were developed in a project on tissue-resident myeloid cells in 2023 and 2024, including a working conference and an in depth discussion among faculty members. The conference on tissue-resident myeloid and histiocytic cells was organized in Vienna (Austria) and took place from June 30 to July 1, 2023. The related consensus project lasted from April 2023 to December 2024. The discussion phase was split into a pre-conference phase (E-mail-based and Web-based: April 2023 until August 2023), the conference, and a post-conference discussion phase (September 2023 to December 2024). The preparation of expert opinions and expert statements were organized in line with available guidelines.^1^

In the final discussion round, the paper-draft was discussed and adjusted based on input provided by all faculty members. Open points were discussed in the faculty (expert group = co-authors) until a clear-cut result (100% of faculty members agreed) was obtained or no consensus was reached. Only those expert opinion statements and definitions that were based on a 100% consensus among all faculty members, were included in the final manuscript version. The final document and its content were approved by all faculty members (all co-authors) before submission.

**Myeloid and Histiocytic Cells Discussed in the Manuscript**

The following cell types were discussed in the conference and reviewed by our faculty members: stem cells and progenitor cells giving rise to myeloid cells, monocytes, monocyte-derived tissue macrophages, normal and neoplastic tissue mast cells and their progenitors, as well as normal and neoplastic dendritic cells (DC), including plasmacytoid DC (pDC). Faculty members reviewed the phenotype and function of these cells in health and disease and discussed how aberrant phenotypes can be exploited to develop diagnostic approaches and therapeutic concepts. In addition, our faculty members reviewed current concepts around neoplastic myeloid states involving these cells, including monocytic leukemias, mastocytosis, histiocytosis and DC neoplasms, with a focus on updated diagnostic criteria, new prognostic and therapeutic concepts, and specific clinical syndromes.

**Role of Tissue-Resident Leukocytes in Inflammation, Atherosclerosis, other Vascular Disorders, and Solid Tumors**

Tissue-resident myeloid cells are variably involved in diverse hypersensitivity reactions, such as hyper-inflammation or anaphylaxis and diverse vascular pathologies.^2-17^ Some of these cells, especially mast cells, are “immediate responders” that can rapidly provide a large panel of vasoactive and pro-inflammatory mediators as well as chemokines and cytokines.^4,8,9,12,15-17^ Moreover, mast cells produce and secrete several repair molecules and proteolytic enzymes that regulate tissue homeostasis.^8,9,12,15-17^ In addition, these cells are a unique source of heparin and of ´uncomplexed´ tissue-type plasminogen activator.^4,17,18^ Therefore, mast cells are considered to participate not only in allergic reactions, fibrosis or tissue damage, but also in tissue repair, fibrinolysis and tissue remodeling.^4,8,15,17^

Monocytes and monocyte-derived macrophages contribute to a number of different pathologic conditions and processes, including infectious diseases, vascular pathologies, and tissue repair.^2,5,6,19^ One interesting aspect is that perivascular macrophages in various organs can interact with a number of other cell types, such as mast cells, eosinophils, lymphocytes, and endothelial cells, and can thereby trigger the initiation and progression of inflammatory and vascular disorders, including atherosclerosis, thromboembolic diseases, infections, or chronic inflammatory disorders (Supplementary Table S3). Several of these cells, such as mast cells, neutrophils, eosinophils, or macrophages produce extracellular traps which support anti-infectious (defense-related) processes but also promote thromboembolic complications and possibly atherosclerosis (Supplementary Table S3). A number of specific cellular interactions between macrophages and other inflammatory cells in the inflamed arterial wall can promote cholesterol accumulation in macrophages and thereby trigger foam cell formation, which in turn promotes the generation of vulnerable atherosclerotic plaques that may rupture and lead to arterial occlusion and tissue infarction.^15,20-22^

Mast cells, monocytes, and granulocytes also act together to counteract and eliminate various infectious diseases, including bacterial, fungal, viral, or parasitic disorders.^23-25^ Some of these cells, such as monocytes, mast cells and their progenitors may serve as a reservoir of certain viruses, including the human immunodeficiency virus (HIV) or SARS-CoV-2.^26-32^ Such virally infected cells may sometimes be eliminated by the virus or by the autologous immune system. In several disease models, the infected local cells also attract additional leukocytes, most probably in an attempt to eliminate the microbes (viruses, bacteria, or fungi) and to clear the affected organs.^23-25^ It is also worth noting that most tissue-resident leukocytes can act as major repair cells that are involved in tissue remodeling and tissue reconstruction after an infectious or other reactive disease process.^8,9,12,15-17^

There are several other reactive disease models where monocytes, tissue-resident macrophages, other histiocytes and/or mast cells, play a major role in disease evolution and progression. Examples include, among others, chronic atopic disorders, autoimmune processes (vasculitis), rheumatologic diseases, and tissue damage caused by environmental toxins. A detailed review of the role of tissue-resident myeloid cells in these diseases is beyond the scope of this manuscript. We refer the interested reader to the available literature.^2-13^

In the context of inflammatory states and cytokine-activation, two types of macrophages have been described.^6,11^ Classically activated macrophages (M1) are activated by interferon-gamma (IFN-γ) and microbial components like lipopolysaccharide. These cells have pro-inflammatory functions and are involved in host defense. By contrast, alternatively activated macrophages (M2) are activated by IL-4, IL-10 and IL-13, and possess distinct cell functions relevant to tissue repair, fibrosis, and immune regulation.

In solid tumors, mast cells and macrophages may also play a role in disease evolution.^33-47^ Although the mechanisms remain largely unknown, several observations suggest that mast cells and tissue macrophages play a suppressive or a supportive role in tumor expansion, progression and invasion, depending on the type and stage of tumor and the experimental model investigated.^36-47^ In local tumor sites, mast cells and macrophages may also increase in number and accumulate and/or show signs of activation.^33-35,39^

A special type of macrophages are tumor-associated macrophages (TAM).^40-47^ These cells belong to the M1 or M2 type of macrophages, depending on the local immune system and the type and stage (aggressiveness) of the tumor (Supplementary Table S4).^40-47^ In early tumor stages, M1 macrophages often predominate. These M1 TAM often exert anti-tumor activity and mediate anti-tumor-directed adaptive immunity by producing pro-inflammatory cytokines such as tumor necrosis factor-alpha (TNF-α), IL-6, IL-12, and IL-1β, which, in turn, triggers M1 polarization.^40,42^ In advanced resistant tumor lesions, M2-polarized TAM often predominate.^40,42^ These cells may still counteract tumor cell growth, but may also suppress tumor adaptive immunity by secreting IL-10, transforming growth factor-beta (TGF-β), and IL-1 receptor antagonist, which leads to tumor progression and contributes to M2 polarization.^40^ Finally, TAM may also interact with other tumor-infiltrating leukocytes and the overall effect of cell-network activation on tumor cell growth and tumor progression depends on multiple tumor-related and patient-specific variables.^38-41^ This is also reflected by varying clinical observations. In fact, depending on the tumor type and tumor stage, the presence and numbers of TAM correlate with a favorable or unfavorable prognosis and outcome.^43-47^

**References**

1. Graham R, Mancher M, Wolman DM, Greenfield S, Steinberg E. Eds, 2011. Institute of Medicine; Board on Health Care Services; Committee on Standards for Developing trustworthy clinical practice guidelines. Clinical practice guidelines we can trust. Washington, DC: National Academies Press. 2011.

2. Davies LC, Jenkins SJ, Allen JE, Taylor PR. Tissue-resident macrophages. *Nat Immunol*. 2013;14(10):986-995.

3. Locati M, Curtale G, Mantovani A. Diversity, mechanisms, and significance of macrophage plasticity. *Annu Rev Pathol*. 2020;15:123-147.

4. Valent P, Akin C, Hartmann K, et al. Mast cells as a unique hematopoietic lineage and cell system: From Paul Ehrlich's visions to precision medicine concepts. *Theranostics*. 2020;10(23):10743-10768.

5. Robinson A, Han CZ, Glass CK, Pollard JW. Monocyte regulation in homeostasis and malignancy. *Trends Immunol*. 2021;42(2):104-119.

6. Mass E, Nimmerjahn F, Kierdorf K, Schlitzer A. Tissue-specific macrophages: how they develop and choreograph tissue biology. *Nat Rev Immunol*. 2023;23(9):563-579.

7. Reste M, Ajazi K, Sayi-Yazgan A, et al. The role of dendritic cells in tertiary lymphoid structures: implications in cancer and autoimmune diseases. *Front Immunol*. 2024;15:1439413.

8. Galli SJ, Grimbaldeston M, Tsai M. Immunomodulatory mast cells: negative, as well as positive, regulators of immunity. *Nat Rev Immunol*. 2008;8(6):478-486.

9. Liu YC, Zou XB, Chai YF, Yao YM. Macrophage polarization in inflammatory diseases. *Int J Biol Sci*. 2014;10(5):520-529.

10. Schultze JL, Schmieder A, Goerdt S. Macrophage activation in human diseases. *Semin Immunol*. 2015;27(4):249-256.

11. Chistiakov DA, Sobenin IA, Orekhov AN, Bobryshev YV. Myeloid dendritic cells: development, functions, and role in atherosclerotic inflammation. *Immunobiology*. 2015;220(6):833-844.

12. Galli SJ, Gaudenzio N, Tsai M. Mast cells in inflammation and disease: recent progress and ongoing concerns. *Annu Rev Immunol*. 2020;38:49-77.

13. Moore KJ, Sheedy FJ, Fisher EA. Macrophages in atherosclerosis: a dynamic balance. *Nat Rev Immunol*. 2013;13(10):709-721.

14. McArdle S, Mikulski Z, Ley K. Live cell imaging to understand monocyte, macrophage, and dendritic cell function in atherosclerosis. *J Exp Med*. 2016;213(7):1117-1131.

15. Kovanen PT. Mast cells as potential accelerators of human atherosclerosis-from early to late lesions. *Int J Mol Sci*. 2019;20(18):4479.

16. Khoury MK, Yang H, Liu B. Macrophage biology in cardiovascular diseases. *Arterioscler Thromb Vasc Biol*. 2021;41(2):e77-e81.

17. Theoharides TC, Valent P, Akin C. Mast cells, mastocytosis, and related disorders. *N Engl J Med*. 2015;373(2):163-172.

18. Sillaber C, Baghestanian M, Bevec D, et al. The mast cell as site of tissue-type plasminogen activator expression and fibrinolysis. *J Immunol*. 1999;162(2):1032-1041.

19. Lavin Y, Mortha A, Rahman A, Merad M. Regulation of macrophage development and function in peripheral tissues. *Nat Rev Immunol*. 2015;15(12):731-744.

20. Galkina E, Ley K. Immune and inflammatory mechanisms of atherosclerosis. Annu Rev Immunol. 2009;27:165-197.

21. Xu JM, Shi GP. Emerging role of mast cells and macrophages in cardiovascular and metabolic diseases. Endocr Rev. 2012;33(1):71-108.

22. Pertiwi KR, de Boer OJ, Mackaaij C, et al. Extracellular traps derived from macrophages, mast cells, eosinophils and neutrophils are generated in a time-dependent manner during atherothrombosis. J Pathol. 2019;247(4):505-512.

23. Echtenacher B, Männel DN, Hültner L. Critical protective role of mast cells in a model of acute septic peritonitis. Nature. 1996;381(6577):75-77.

24. Malaviya R, Ikeda T, Ross E, Abraham SN. Mast cell modulation of neutrophil influx and bacterial clearance at sites of infection through TNF-alpha. Nature. 1996;381(6577):77-80.

25. Féger F, Varadaradjalou S, Gao Z, et al. The role of mast cells in host defense and their subversion by bacterial pathogens. Trends Immunol. 2002;23(3):151-158.

26. Bannert N, Farzan M, Friend DS, et al. Human mast cell progenitors can be infected by macrophagetropic human immunodeficiency virus type 1 and retain virus with maturation in vitro. J Virol. 2001;75(22):10808-10814.

27. Sundstrom JB, Ellis JE, Hair GA, et al. Human tissue mast cells are an inducible reservoir of persistent HIV infection. Blood. 2007;109(12):5293-5300.

28. McNamara LA, Collins KL. Hematopoietic stem/precursor cells as HIV reservoirs. Curr Opin HIV AIDS. 2011;6(1):43-48.

29. Calado M, Pires D, Conceição C, et al. Cell-to-cell transmission of HIV-1 and HIV-2 from infected macrophages and dendritic cells to CD4+ T lymphocytes. Viruses. 2023;15(5):1030.

30. Woottum M, Yan S, Sayettat S, et al. Macrophages: Key Cellular Players in HIV Infection and Pathogenesis. Viruses. 2024;16(2):288.

31. Banga R, Perreau M. The multifaceted nature of HIV tissue reservoirs. Curr Opin HIV AIDS. 2024;19(3):116-123.

32. Patterson BK, Francisco EB, Yogendra R, et al. Persistence of SARS CoV-2 S1 protein in CD16+ monocytes in post-acute sequelae of COVID-19 (PASC) up to 15 months post-infection. Front Immunol. 2022;12:746021.

33. Takanami I, Takeuchi K, Naruke M. Mast cell density is associated with angiogenesis and poor prognosis in pulmonary adenocarcinoma. Cancer. 2000;88(12):2686-2692.

34. Ribatti D. Mast cells and macrophages exert beneficial and detrimental effects on tumor progression and angiogenesis. Immunol Lett. 2013;152(2):83-88.

35. Sammarco G, Varricchi G, Ferraro V, et al. Mast cells, angiogenesis and lymphangiogenesis in human gastric cancer. Int J Mol Sci. 2019;20(9):2106.

36. Longo V, Catino A, Montrone M, et al. Controversial role of mast cells in NSCLC tumor progression and angiogenesis. Thorac Cancer. 2022;13(21):2929-2934.

37. Molfetta R, Paolini R. The controversial role of intestinal mast cells in colon cancer. Cells. 2023;12(3):459.

38. Kwantwi LB. Exosome-mediated crosstalk between tumor cells and innate immune cells: implications for cancer progression and therapeutic strategies. J Cancer Res Clin Oncol. 2023;149(11):9487-9503.

39. Attramadal CG, Kumar S, Gao J, et al. Low mast cell density predicts poor prognosis in oral squamous cell carcinoma and reduces survival in head and neck squamous cell carcinoma. Anticancer Res. 2016;36(10):5499-5506.

40. Allavena P, Sica A, Garlanda C, Mantovani A. The Yin-Yang of tumor-associated macrophages in neoplastic progression and immune surveillance. Immunol Rev. 2008;222:155-161.

41. Gordon SR, Maute RL, Dulken BW, et al. PD-1 expression by tumour-associated macrophages inhibits phagocytosis and tumour immunity. Nature. 2017;545(7655):495-499.

42. Boutilier AJ, Elsawa SF. Macrophage polarization states in the tumor microenvironment. Int J Mol Sci. 2021;22(13):6995.

43. Cencini E, Fabbri A, Sicuranza A, et al. The role of tumor-associated macrophages in hematologic malignancies. Cancers (Basel). 2021;13(14):3597.

44. Mei J, Xiao Z, Guo C, et al. Prognostic impact of tumor-associated macrophage infiltration in non-small cell lung cancer: A systemic review and meta-analysis. Oncotarget. 2016;7(23):34217-34228.

45. Chen YL. Prognostic significance of tumor-associated macrophages in patients with nasopharyngeal carcinoma: A meta-analysis. Medicine (Baltimore). 2020;99(39):e21999.

46. Ma Y, Sun Y, Guo H, Yang R. Tumor-associated macrophages in bladder cancer: roles and targeted therapeutic strategies. Front Immunol. 2024;15:1418131.

47. Lin X, Zhan J, Guan Z, et al. Clinicopathologic and prognostic significance of tumor-associated macrophages in cervical cancer: a systematic review and meta-analysis. Clin Transl Oncol. 2025;27(1):351-362.

48. Arber DA, Orazi A, Hasserjian RP, Borowitz MJ, Calvo KR, Kvasnicka HM, et al. International Consensus classification of myeloid neoplasms and acute leukemias: integrating morphologic, clinical, and genomic data. Blood. 2022;140(11):1200-1228.

49. Campo E, Jaffe ES, Cook JR, Quintanilla-Martinez L, Swerdlow SH, Anderson KC, et al. The International Consensus Classification of mature lymphoid neoplasms: a report from the clinical advisory committee. Blood. 2022;140(11):1229-1253.

50. Valent P, Orazi A, Savona MR, Patnaik MM, Onida F, van de Loosdrecht AA, et al. Proposed diagnostic criteria for classical chronic myelomonocytic leukemia (CMML), CMML variants and pre-CMML conditions. Haematologica. 2019;104(10):1935-1949.

51. Valent P, Horny HP, Escribano L, Longley BJ, Li CY, Schwartz LB, et al. Diagnostic criteria and classification of mastocytosis: a consensus proposal. Leuk Res. 2001;25(7):603-625.

52. Valent P, Akin C, Hartmann K, Alvarez-Twose I, Brockow K, Hermine O, et al. Updated diagnostic criteria and classification of mast cell disorders: a consensus proposal. Hemasphere. 2021;5(11):e646.

53. McClain KL, Bigenwald C, Collin M, Haroche J, Marsh RA, Merad M, et al. Histiocytic disorders. Nat Rev Dis Primers. 2021;7(1):73.

54. Henter JI, Sieni E, Eriksson J, Bergsten E, Hed Myrberg I, Canna SW, et al. Diagnostic guidelines for familial hemophagocytic lymphohistiocytosis revisited. Blood. 2024;144(22):2308-2318.

**Supplementary Tables**

Supplementary Table S1

**Potential physiologic functions of tissue-resident myeloid cells and their contribution to tissue integrity, defense, and tissue homeostasis**

-----------------------------------------------------------------------------------------------------------------

Cell type Putative functions in healthy tissues and reactive states

-----------------------------------------------------------------------------------------------------------------

Monocytes Recognition and clearing (by phagocytosis) of invading pathogens,

mobilization and attraction of additional leukocytes (phagocytes)

through the production and release of cytokines, chemokines, and other relevant pro-inflammatory substances*; differentiation into tissue-specific macrophages.

Macrophages Recognition and clearing (by phagocytosis) of invading pathogens,

mobilization and attraction of additional leukocytes (phagocytes)

through production and release of cytokines, chemokines, and

other relevant pro-inflammatory substances*;

regulation of vascular processes, coagulation and fibrinolysis,

tissue repair following inflammation and tissue damage.

Dendritic cells Recognition of pathogens and antigen presentation with

subsequent clearing (by phagocytes) of invading pathogens;

Mobilization and attraction of additional leukocytes (phagocytes)

through cytokine and chemokine production and release.

Mast cells Regulation of endothelial cell function and permeability, as well

as the influx of leukocytes from the peripheral blood into tissues;

production, storage and rapid release of pro-inflammatory and

vasoactive mediators and cytokines, including mediators of acute

inflammation and anaphylaxis;** regulation of local fibrinolysis

and tissue repair after inflammation and tissue damage;***

degradation and neutralization of venoms and other exogenous

pathogens by specific proteolysis (tryptases, chymases).

-----------------------------------------------------------------------------------------------------------------

*Clinically relevant cytokines/chemokines and other compounds produced by monocytes and macrophages include, among others, interleukin-1 (IL-1), IL-4, IL-6, IL-10, IL-15, IL-17, vascular endothelial growth factor (VEGF), various leukocyte-activating and attracting chemokines, platelet-activating factor (PAF), tissue factor, tissue-type plasminogen activator (tPA), urokinase and plasminogen activator inhibitors. **Specific mediators of acute inflammation and anaphylaxis produced in mast cells include histamine, prostaglandin D2, certain leukotrienes, and cytokines such as tumor necrosis factor (TNF). ***Tissue mast cells are a unique source of heparin and pro-fibrinolytic agents, such as uncomplexed tissue type plasminogen activator (tPA), and various (additional) repair molecules such as IL-1 and VEGF.

Supplementary Table S2

**Monocytic, histiocytic, dendritic, and mast cell neoplasms recognized by the international consensus classification (ICC) consortium in 2022***

-----------------------------------------------------------------------------------------------------------------

Mastocytosis

- Cutaneous mastocytosis (CM)

- Systemic mastocytosis (SM) including special variants such as:

- SM with an associated myeloid neoplasm (SM-AMN)

Myelodysplastic/myeloproliferative neoplasms with prominent monocytosis

 - Chronic myelomonocytic leukemia (CMML)

 - Clonal cytopenia with monocytosis of undetermined significance**

 - Clonal monocytosis of undetermined significance**

Pediatric and/or germline mutation-associated disorders with monocyte involvement

 - Juvenile myelomonocytic leukemia

 - Juvenile myelomonocytic leukemia-like neoplasms

Acute myeloid leukemia (AML): no particular monocytic or monoblastic (monocyte-
 lineage) AML variant is reported by the ICC

Blastic plasmacytoid dendritic cell neoplasm(s) (BPDCN)

Other histiocytic and dendritic cell neoplasms

-----------------------------------------------------------------------------------------------------------------

*The classification was published in Arber et al, Blood 2022;140:1200-1228 and Campo et al, Blood 2022;140:1229-1253.^48,49^ A more detailed description of the classification is shown in other supplementary Tables (see down below in this supplement).

**These clonal conditions are no overt neoplasms but may develop into an overt neoplasm, such as CMML or AML.

Supplementary Table S3

**Roles of macrophages and mast cells in atherosclerosis and vascular repair after a thromboembolic event**

--------------------------------------------------------------------------------------------------------

Cells Roles in Atherosclerosis Roles in vascular repair

--------------------------------------------------------------------------------------------------------

Macrophages Generation of pro-inflammatory Generation and release of

and pro-atherogenic cytokines repair-promoting cytokines

(IL-1, TNF, GM-CSF, others) (VEGF, PDGF, others)

Foam cell formation Induction of neo-angiogenesis

Plaque formation and

plaque rupture

Mast cells Generation of pro-inflammatory Generation and release of

and pro-atherogenic cytokines repair-promoting cytokines

(TNF, others) (VEGF, PDGF, HGF, others)

Generation of other pro- Production of heparin

inflammatory mediators Production of tryptases,

(histamine, PGD2) chymases and tPA

Plaque rupture Induction of neo-angiogenesis

(heparin+tPA)

Thrombus formation and

thrombus stabilization

(C5a+PAI-1)

----------------------------------------------------------------------------------------------------------------

Abbreviations: IL-1, interleukin-1; TNF, tumor necrosis factor; GM-CSF, granulocyte/macrophage colony-stimulating factor; VEGF, vascular endothelial growth factor; PDGF, platelet-derived growth factor; HGF, hepatocyte growth factor; PGD2, prostaglandin D2, tPA, tissue-type plasminogen activator; C5a, complement factor 5a; PAI-1, plasminogen activator inhibitor 1.

Supplementary Table S4

**Impact of tumor-associated M1 macrophages (M1 TAM) and M2 macrophages (M2 TAM) in the evolution, progression and resistance of tumor cells**

-----------------------------------------------------------------------------------------------------------------

Tumor stages and progression Role of M1 TAM or M2 TAM

-----------------------------------------------------------------------------------------------------------------

Early tumor stages M1 TAM have major antigen-presenting capacity

M1 TAM counteract tumor expansion

M1 TAM can directly attack and kill certain

tumor cells (direct lysis, TNF-mediated lysis,

and antibody or T cell-dependent killing)

M1 TAM mediate anti-tumor adaptive immunity M1 TAM lack PD-1

Later tumor stages M2 TAM have poor antigen-presenting capacity

M2 TAM suppress tumor adaptive immunity

M2 TAM suppress tumor immune responses

M2 TAM display PD-1 and may thereby

mediate T cell resistance of tumor cells

M2 TAM promote tumor angiogenesis

M2 TAM promote metastatic progression

-----------------------------------------------------------------------------------------------------------------Abbreviations: PD-1, programmed death receptor-1.

Supplementary Table S5

**Morphologic delineation of blast cells and monocytic cells in patients with myelomonocytic and monocytic AML or CMML***

---------------------------------------------------------------------------------------------------------------------------

Nuclear Size relative to

Cell Type shape Chromatin Cytoplasm mature monocytes

---------------------------------------------------------------------------------------------------------------------------

Blast cells:

Myeloblast Round/oval Fine with Basophilic, Smaller

nucleoli rare or no

granules

Monoblast Round/oval Delicate / Basophilic, Large

lace-like, rare azurophilic (20-30 µM)

nucleoli granules

Promonocyte Convoluted/ Delicate / Variably basophilic, Large

indented** lace-like, variable azurophilic

nucleoli granules

Monocytic cells:

Abnormal/immature Convoluted/ More condensed, Intermediate Smaller

monocyte indented rare nucleoli basophilic***

Mature monocyte Lobulated/ Condensed, Grey or pinkish =

indented no nucleoli with occasional

azurophilic

granules

and vacuoles

---------------------------------------------------------------------------------------------------------------------------

*A detailed description of morphologic features and criteria of neoplastic monocytic cells has been described in Valent et al. Haematologica 2019;104:1935-1949.^50^

**The most important delineating feature discriminating promonocytes from monoblasts.

***Less basophilic than promonocytes and more basophilic than mature monocytes.

Supplementary Table S6

**Major and Minor Criteria of Systemic Mastocytosis (SM Criteria)**

-----------------------------------------------------------------------------------------------------------------

Major criterion: Multifocal dense infiltrates of mast cells (>15 mast cells

in aggregates) in bone marrow biopsies and/or in sections of

other extracutaneous organ(s)

Minor criteria:

a. >25% of all mast cells are atypical cells (type I or type II) on

bone marrow smears or are spindle-shaped in mast cell infiltrates

detected in sections of BM or other extra-cutaneous organs*

b. KIT-Activating *KIT* point mutation(s) at codon 816 or other critical

regions of *KIT*** in bone marrow or another extra-cutaneous organ

c. Mast cells in bone marrow, blood or other extra-cutaneous organs

express one or more of: CD2 and/or CD25 and/or CD30***

d. Baseline serum tryptase concentration >20 ng/ml (in the presence of an associated myeloid neoplasm, an elevated tryptase does not count as SM criterion). In the case of a known hereditary alpha tryptasemia (HαT), the tryptase level should be adjusted.****

If at least 1 major and 1 minor or 3 minor SM criteria are fulfilled

→ the diagnosis is systemic mastocytosis = SM

-----------------------------------------------------------------------------------------------------------------

*In tissue sections, an abnormal mast cell morphology counts when observed in compact and diffuse mast cell infiltrates. However, the spindle-shaped form does not count as an SM criterion when mast cells are lining vascular cells, fat cells, nerve cells or the endosteum layer. In the bone marrow smear, an atypical morphology of mast cells does not count as SM criterion when mast cells are located in or adjacent to bone marrow particles.

**Any type of *KIT* mutation counts as minor SM criterion when published solid evidence for its transforming behavior is available, such as mutations in *KIT* codons 417, 501-509, 522, 557-560, 642, 654, 799, 816, 820, or 822.

***All three markers fulfil this minor SM criterion when expression can be confirmed by flow cytometry and/or immunohistochemistry.

****A straightforward way to adjust to divide the basal tryptase level by 1 plus the extra copy numbers of the alpha tryptase gene.

The diagnostic criteria were first published in Valent et al, Leuk Res 2001;25:603-625, and more recently in slightly modified form in Valent et al, Hemasphere 2021;5:e646.^51,52^

Supplementary Table S7

**WHO Classification of Mastocytosis and Prognostic Impact**

----------------------------------------------------------------------------------------------------------------

Risk of Risk of

Variant and Sub-Variant Progression* Anaphylaxis

----------------------------------------------------------------------------------------------------------------

Cutaneous Mastocytosis (CM)

Maculopapular CM Very Low** Intermediate

Diffuse CM (DCM) Very Low High

Mastocytoma of Skin Very Low Low

Systemic Mastocytosis (SM)

Bone Marrow Mastocytosis (BMM)*** Very Low High

Indolent SM (ISM) Low Intermediate to High

Smoldering SM (SSM) Intermediate Intermediate

SM with an AHN (SM-AHN) High Low

Aggressive SM (ASM) High Low

Mast Cell Leukemia (MCL) Intermediate Low

Mast Cell Sarcoma (MCS) Very High Low

----------------------------------------------------------------------------------------------------------------

*Progression into a higher-grade mast cell neoplasm or from SM into SM-AHN.

**Although the risk of progression in CM is very low, a few patients with MPCM may develop SM.

***In the latest update of the WHO classification, BMM is regarded as a provisional entity and subset of ISM. Abbreviations: WHO, World Health Organization; SM, systemic mastocytosis; AHN, associated hematologic neoplasm.

Supplementary Table S8

**Proposed Clinical Classification of Histiocytic Disorders with Diagnostic Criteria and Typical Clinical Features and Clinical Impact***

-----------------------------------------------------------------------------------------------------------------

**Cutaneous Disease = C-Group** (major clinical impact: cutaneous lesions)

1. Cutaneous non-Langerhans-Cell-Histiocytosis (non-LCH)

 - Non-XG family: includes cutaneous RDD

 - XG family: includes JXG

2. Cutaneous non-LCH with major systemic component

**Hemophagocytosis = H-Group** (major clinical impact: hemophagocytosis)

1. Primary/inherited form of hemophagocytic lymphohistiocytosis (HLH)

(monogenic inherited form of disease)

2. Secondary HLH (non-inherited HLH)

3. HLH with uncertain etiology

**Langerhans cell disorders = L-Group** (impact: Langerhans cell accumulation)

1. Langerhans cell histiocytosis (LCH)

2. Erdheim-Chester disease (ECD)

3. Mixed LCH/ECD

**Malignant histiocytosis = H-Group** (major clinical impact: malignant course)

1. Primary malignant histiocytoses:

- Diagnostic criteria for a histiocytosis are fulfilled by WHO or ICC criteria

- The disease shows a progressive (malignant/aggressive) clinical course

- WHO or ICC criteria for another hematopoietic neoplasm are not met

2. Secondary malignant histiocytosis:

- Diagnostic criteria for a histiocytosis are fulfilled by WHO or ICC criteria

- The disease shows a progressive (malignant/aggressive) clinical course

- WHO or ICC criteria for another hematopoietic neoplasm are fulfilled

**Rosaí Dorfman disease (RDD) = R-Group** (clinical impact: RDD)

1. Sporadic RDD

- Classic RDD

- Extranodal RDD

- RDD with neoplasm or immune disease

- Unclassified

2. Familial RDD

-----------------------------------------------------------------------------------------------------------------

*A similar classification has been presented by McClain et al., Nat Rev Dis Primers. 2021;7:73 and (in revised form) by Henter et al., Blood. 2024;144(22):2308-2318.^53,54^

Supplementary Table S9

**Histiocytic and Dendritic Cell Neoplasms Defined in The International Consensus Classification (ICC) Proposal 2022**

-----------------------------------------------------------------------------------------------------------------

1. Variants reported in the report on mature lymphoid neoplasms in 2022

(Campo et al, Blood 2022;140:1229-1253)^49^

 Histiocytic sarcoma

 Langerhans cell histiocytosis

 Langerhans cell sarcoma

 Indeterminate dendritic cell histiocytosis (wording: histiocytosis instead of tumor)*

 Interdigitating dendritic cell sarcoma

 ALK-positive histiocytosis (new category defined by the WHO and ICC in 2022)*

 Disseminated juvenile xanthogranuloma

 Erdheim-Chester disease

 Rosai-Dorfman-Destombes disease (new category; regarded as neoplasm)*

 Follicular dendritic cell sarcoma

 Fibroblastic reticular cell sarcoma (wording changed from tumor to histiocytosis)*

 Epstein-Barr virus–positive inflammatory follicular dendritic cell/

fibroblastic reticular cell tumor (wording changed from sarcoma to tumor)*

2. Variants reported in the WHO description of myeloid neoplasms in 2022

(Arber et al, Blood 2022;140:1200-1228)^48^

Blastic plasmacytoid dendritic cell neoplasm

-----------------------------------------------------------------------------------------------------------------

*In these categories, changes compared to the WHO classification 2016 have been reported by the authors. Some of these histiocytic neoplasms are newly integrated in the classification of both the ICC and WHO.

Supplementary Table S10

**Proposed Diagnostic (Clinical) Criteria to Diagnose Hemophagocytic Lymphohistiocytosis (HLH) in Daily Practice***

--------------------------------------------------------------------------------------------------------

1. Fever (> 38°C, persistent, not attributable to another etiology)

2. Splenomegaly (palpable)

3. Cytopenia in at least two of the following cell lineages:

- hemoglobin < 9 g/dL (< 10 g/dL in neonates)

- platelets (< 100,000/µL)

- neutrophils (< 1,000/µL)

4. Hypertriglyceridemia (> 265 mg/dl; equals > 3 mmol/L) or

Hypofibrinogenemia (< 150 mg/dl)

5. Hyperferritinemia (> 500 g/L); optimized threshold in adults

with suspected hematologic disorder: 1,000g/L

6. Soluble CD25 serum levels (sCD25) > 2,400 U/mL; optimized
 threshold in adults with suspected hematologic disorder: 3,900 U/ml

7. Hemophagocytosis in bone marrow, spleen, lymph

nodes or liver, as observed in histological sections

--------------------------------------------------------------------------------------------------------

*The Histiocyte Society established diagnostic criteria for HLH for use in clinical trials (McClain et al., Nat Rev Dis Primer 2021;7:73)^53^ which have been adapted in 2024 (HLH-2024) through deletion of NK-cell testing (Henter et al, Blood 2024;144:2308-2318).^54^ These criteria are now also recommended for use in daily practice. A clinical syndrome of HLH can be diagnosed if at least five out of seven clinical and laboratory criteria depicted above are present. In adult patients in workup for hematologic disorders with hyperinflammation or fever of unknown origin, the optimized HLH index (OHI) provides improved threshold values for Ferritin and sCD25.

A genetic condition predisposing to HLH can be diagnosed based on the presence of a pathogenic variant in a known causative gene (see text in this article). However, a genetic defect alone does not imply that the patient has overt HLH unless HLH criteria are fulfilled. Apart from genetic predisposition, various hematologic neoplasms, including histiocytic and dendritic cell neoplasms (defined in part by somatic mutations) may also predispose to the development of HLH (somatic predisposition).

Supplementary Table S11

**Specific Clinical Syndromes Reported in Patients Suffering from Disorders of Tissue-Resident Myeloid Cells***

--------------------------------------------------------------------------------------------------------

Typical underlying conditions*

-------------------------------------------------------------------

Syndrome Reactive conditions Neoplasm(s)

--------------------------------------------------------------------------------------------------------

Hemophagocytic Rheumatologic disease Myeloid neoplasm,

lymphohistiocytosis Infectious disease lymphoma (NHL), or

(HLH)** histiocytic/dendritic

cell neoplasm

Mast cell activation IgE-dependent allergy, Mastocytosis

syndrome (MCAS) intolerance, or toxic- (CM or SM)

allergic reaction

Hypereosinophilic Reactive hypereosinophilia Eosinophilic

syndromes (HES)*** (worm infections, other leukemia, myeloid

infections, allergy, T cell or lymphoid

neoplasms) neoplasm with

clonal eosinophilia

Tumor-associated - Myeloid neoplasms,

coagulopathy (DIC) especially monocytic

with consumption** and monoblastic AML

but also in histiocytic

neoplasms (+/-HLH)**

Tumor-lysis syndrome** - Various myeloid,

histiocytic/dendritic

cell or mast cell neoplasms (occurs

during therapy)**

--------------------------------------------------------------------------------------------------------

*In addition to these conditions, each syndrome may also have a genetic background (familial forms) or may occur as an idiopathic form (McClain et al., Nat Rev Dis Primer 2021;7:73; Henter et al, Blood 2024;144:2308-2318).^53,54^ HLH may be associated with DIC and may also develop (manifest or aggravate) during anti-neoplastic therapy). In all these patients, bleeding may occur as a result of thrombocytopenia and/or a fast decrease (by endogenous consumption) of coagulation-promoting factors (fibrinogen and others). ***HES is defined by hypereosinophilia (HE=eosinophils >1500/µL blood) and HE-induced organ damage. In many of these patients, thromboembolic events are recorded.
